# The Patients' Perception of Prodromal Symptoms Before the Initial Diagnosis of Parkinson's Disease

**DOI:** 10.1002/mds.23499

**Published:** 2011-03-02

**Authors:** Alexandra Gaenslen, Irene Swid, Inga Liepelt-Scarfone, Jana Godau, Daniela Berg

**Affiliations:** 1 Department of Neurodegeneration, Hertie Institute of Clinical Brain ResearchTübingen, Germany; 2 German Center for Neurodegenerative Diseases, University of TübingenGermany (DZNE)

**Keywords:** Parkinson's disease/parkinsonism, prodromal phase, Braak staging

## Abstract

**Background:**

Before the occurrence of motor symptoms permits the clinical diagnosis of Parkinson's disease (PD), about or even more than 50% of the dopaminergic neurons of the substantia nigra have degenerated. This time be called the prodromal phase of PD.

**Objective:**

To evaluate the time span from onset of first prodromal symptoms to the initial diagnosis of PD as well as the order of symptom occurrence.

**Methods:**

Retrospective study of 93 consecutively interviewed PD patients without dementia and 93 sex and age matched controls free of neurodegenerative disorders. A standardized in-house telephone worksheet assessing 19 nonmotor and six early motor signs was used.

**Results:**

A total of 98.8% of all patients interviewed reported to have experienced prodromal symptoms prior to receiving the initial diagnosis of PD. Patients noticed an average of 7.6 different symptoms during this time interval. The mean time span between the recalled onset of any one symptom and PD diagnosis was 10.2 years. In both groups, the course of prodromal sign onset was associated with early neuropathological disease stages proposed by Braak.

**Outlook:**

These retrospectively gathered data confirm the existence of a long prodromal phase for PD that is consistent with neuropathological staging. A standardized questionnaire assessing such early symptoms may be helpful in identifying subjects at high risk for PD while they are still in the prodromal phase of the disorder. © 2011 Movement Disorder Society

When Parkinson's disease (PD) is noticed by patients and diagnosed by clinicians, about or even more than 50% of the dopaminergic cells in the substantia nigra (SN) are reported to have degenerated.^1^ The time span between the onset of neurodegeneration and manifestation of the typical motor symptoms is referred to as premotor or prodromal phase of PD.^2^–^4^ The duration of this phase prior to initial diagnosis of PD is still a matter of debate^5^ and has been estimated to last for years^6^ or even decades.^4^,^7^,^8^

This premotor phase, however, is not clinically silent. Premotor symptoms are known to antecede the typical clinical symptom constellation (akinesia plus rigidity and/or tremor with difference in severity of side affection) leading to the clinical diagnosis (UK Brain Bank criteria).^9^

Early symptoms are probably caused by affection of lower brainstem and spinal cord areas as well as the olfactory bulb. The appearance of these symptoms has been suggested to be accompanied or caused by the presence Lewy pathology at these sites before the SN is involved to such a degree that motor signs permit the diagnosis of PD.^10^ A number of additional studies have provided evidence that constipation^11^ and other signs of autonomic dysfunction,^2^,^12^ hyposmia,^13^ pain,^3^ REM-sleep-behavior-disorder (RBD)^14^,^15^ neuropsychiatric complaints (anxiety, depression), and minor cognitive deficits^16^–^18^ may also be subsumed under this group. Mild motor signs, such as an asymmetric arm swing, which cannot unambiguously be classified as symptoms of PD, when occurring singularly, may be attributable to the progressive cell loss of the SN that is not severe enough to result in the classical clinical presentation of PD.^9^

Because both nonmotor and slight unspecific motor symptoms can precede PD diagnosis according to published diagnostic criteria, we refer to this period in the following as the prodromal phase.

Many PD patients seem to notice nonmotor and early motor symptoms, as evidenced by the fact that they go to see general practitioners with psychological or painful musculoskeletal complaints more often than controls, with an increasing rate of consultations 3–6 years prior to initial diagnosis.^19^–^22^ Moreover, in a prospective study, it could be shown that early unclear motor signs, including stiffness, tremor of the extremities or head, slowed movements, feeling of lost balance and/or falls were reported by more than 70% of those individuals in whom PD was diagnosed after a mean follow-up of 5.8 years.^23^

Because the chronology of such nonmotor and slight motor symptoms is unknown, this retrospective study aimed to characterize the prodromal period more clearly by assessing patients' perception of both these signs.

## Methods

### Patients and Recruitment

For this study, which was approved by the local ethical committee, we questioned 93 consecutive patients with idiopathic PD (according to UKBB clinical diagnostic criteria^24^), who were seen as outpatients by movement disorder specialists at the University of Tübingen, Department of Neurodegeneration within a 12-months period prior to the telephone interview. Under the same conditions, 93 gender and age-matched control persons free of neurodegenerative disorders were interviewed. Inclusion criteria for PD patients were Hoehn & Yahr stage 1–3 and fluency in German language. Exclusion criteria were signs of dementia according to the DSM-IV criteria assessed by an experienced clinician and a score ≤ 26 in the Mini-Mental-State-Examination,^25^ evidence of atypical Parkinsonian syndromes, severe head trauma or other central neurological disorders. The control individuals were recruited from a large sample of an epidemiological population-based study of 812 persons (n = 40)^26^ and from a second, still unpublished cross-sectional study of 555 persons (n = 53). For both cross-sectional studies, volunteers (>50 years of age) free of neurodegenerative disorders were recruited with the help of advertisements placed in local newspapers. All patients and controls were invited by letter to participate in a telephone interview of ∼45 min duration during which they would be questioned about the occurrence of nonmotor and early nonspecific motor symptoms.

### Telephone Interview

The telephone interview was performed in a structured fashion using an in-house standardized worksheet. The interview consisted of 25 specific questions designed to assess presence of 19 nonmotor and six early motor symptoms in the patient history.

The following 19 nonmotor symptoms were subdivided into six categories:

visual abnormalities: (1) disturbance of colour vision;
sleep disturbances: signs of RBD, i.e., (2) crying during sleep, (3) nightmares or (4) vivid dreams, (5) limb movements during sleep or other sleep disturbance e.g., (6) problems with staying asleep or (7) falling asleep;^27^
(8) anosmia or hyposmia;
autonomic dysfunction—(9) constipation, (10) increased sweating, (11) seborrhoea and (12) orthostatic dizziness;
psychiatric complaints, including (13) anxiety, (14) moodiness, (15) depression, or (16) lack of motivation in the performance of daily activities (apathy);
cognitive impairment or abnormalities, including (17) problems to recall names and other essential information, (18) forgetfulness and finding of words or (19) slowed thinking (bradyphrenia).

The six additional questions for evaluating early motor slowing (hereafter referred to as “early motor signs”) included: (20) hypophonia, (21) dysarthria, (22) sialorrhoea as a result of reduced swallowing, (23) slowing of fine hand movements, (24) general slowing—bradykinesia and (25) unilaterally reduced arm swing.

For each sign, participants were directed to respond with “yes” or “no.” For affirmative answers, participants were asked to report the time of the first perception in years before the diagnosis of PD was made (patients) or the timespan in years (controls). For PD patients, only the symptoms reported to have started before the diagnosis of PD were counted. The anticipated prodromal time span therefore was the time of nonmotor or nonspecific symptom onset to initial diagnosis for the patients, for the controls it was the time span of symptom onset to the interview.

### Statistics

Statistical analyses were performed with SPSS 17.0 for Windows SPSS (Chicago, IL, III). For the group comparison according to the occurrence of characteristics or categorical data Chi-square-test was applied (*P* < 0.05). For variables with normal distribution (Kolmogoroff Smirnoff Test *P* > 0.05) the Student's *t*-test was applied, for all other variables the Mann-Whitney U-Test was performed. For analysis of the mean number of signs in different age ranges (Table 2), Bonferoni-correction for multiple testing was applied (*P* = 0.05/5 = 0.01).

**TABLE 2:** Mean number of signs for different age ranges (retrospectively gathered data)

	< 40 years	41–50 years	51–60 years	61–70 years	> 71 years
PD					
n	93	82	55	13	13
Mean	0.72	1.9	3.6	5.3	5.9
SD	1.73	2.4	3	3.6	3.5
Range	0–10	0–9	0–14	0–10	2–14
Controls					
n	93	92	78	34	34
Mean	0.26	0.3	1.2	2.3	2.8
SD	0.64	0.7	1.3	2	2
Range	0–3	0–3	0–6	0–6	0–6
*P*-value (Mann-Whitney-test)	0.175	<0.001	<0.001	0.01	<0.001

Bonferoni correction for multiple comparisons: significance at *P* < 0.01.

### Demographical Data

Mean age of PD patients was 67.9 years ± 7.3 years, mean age of controls was 67.7 ± 7.2 years, (*P* = 0.9). In both groups, 33 subjects (35.5%) were female (*P* = 1.0). In the group of the PD patients 47.3% were H&Y stage 1.0, 35.5%—2.0 and 17.2%—3.0. 25.8% patients had a rigid-akinetic type of PD, in 28% a tremor-dominant and in 46.2% a mixed form of PD was diagnosed. The mean duration from diagnosis of PD to interview was 5.9 ± 5.7 years.

## Results

### Prodromal Symptoms in PD Patients and Controls

98.9% of the PD patients and 97.4% of the controls responded affirmative to at least one question regarding prodromal symptoms. 92.5% of the PD patients and 90.3% of the controls reported one or more nonmotor sign(s), and 95.7% of the PD patients as opposed to 37.6% of the controls experienced some early nonspecific or slight motor symptoms (Table 1).

**TABLE 1:** Number of prodromal, nonmotor and early motors signs in PD patients and controls

Number of signs out of 25		All prodromal	Nonmotor	Early motor
PD-patients, n = 93	Mean	7.6	4.8	2.8
	SD	4	3.3	1.3
	Range	0–16	0–13	0–6
Controls, n = 93	Mean	4	3.5	0.5
	SD	2.8	2.4	0.8
	Range	0–13	0–10	0–4
*P*-value (Mann-Whitney-test)		<0.0001	0.017	<0.0001

**FIG. 1:**
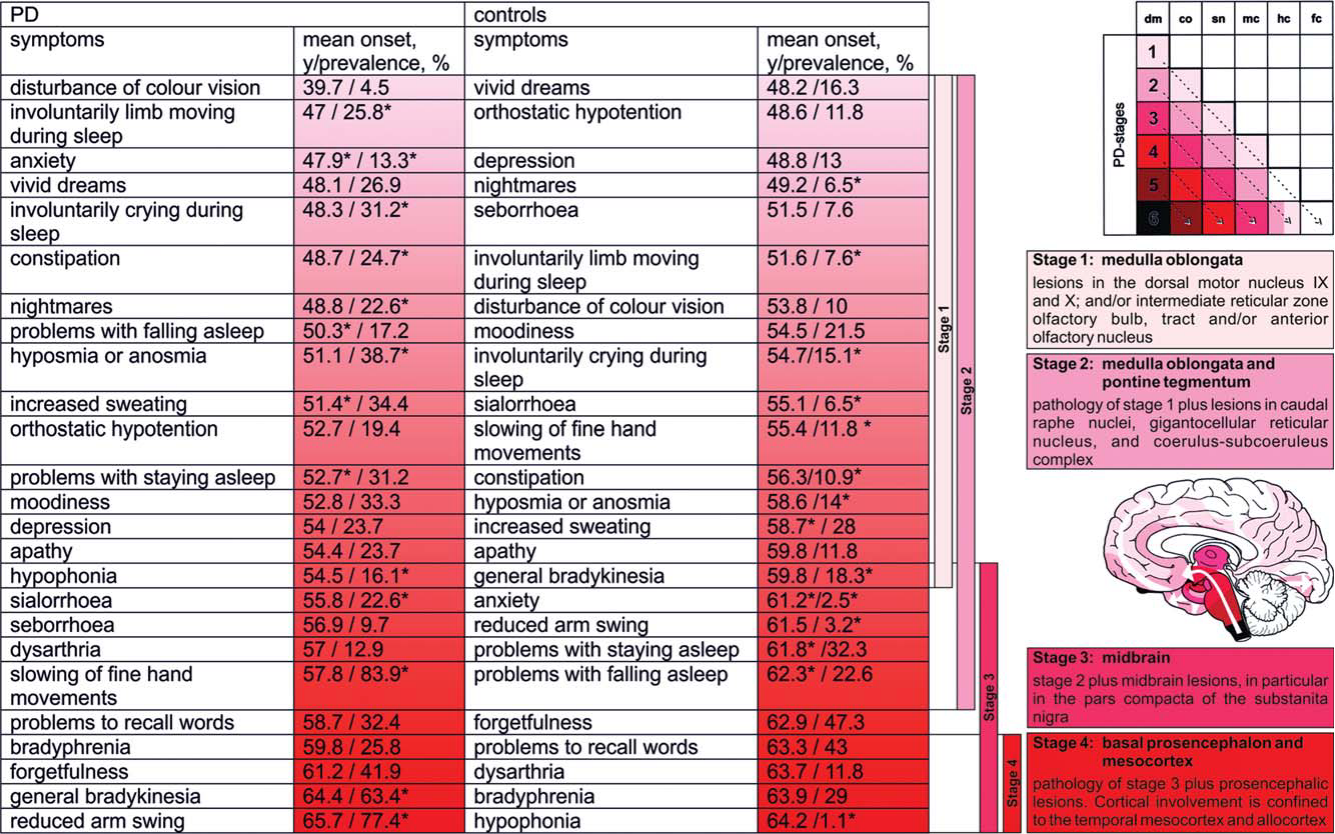
Illustration of hypothesis: Reported symptoms of PD patients and controls in the prodromal phase of PD with regard to mean age (years, y) at onset (modified from^10^ and^28^). The schema of stages of PD and image of brain are used with friendly permission of Braak et al.^10^ * *P*-value for the test < 0.05. [Color figure can be viewed in the online issue, which is available at wileyonlinelibrary.com.]

Six of 19 (31.6%) nonmotor and five of six (83.3%) early motor symptoms were reported significantly more often by PD patients than by controls (Fig. 1).

The age of symptom onset did not significantly differ for patients and controls with exception of three symptoms (increased sweating, problems with falling and with staying asleep, (Fig. 1). But taking all signs together, there was a significant group difference in the age of onset of any first sign, with earlier onset in the group of PD patients at 51.2 (SD 11.6) years compared with controls in whom the recalled age of first perception was 57.2 years (SD 10.3), *P* < 0.001.

In each age range starting from 41 years, PD patients reported significantly more prodromal signs than controls (Table 2).

### Time Course of Prodromal Symptoms in PD

The mean duration of the evaluated premotor and early motor symptoms before the diagnosis of PD was 10.6 (SD 8.1) and 3.3 years (SD 2.7). The first signs noticed by the patients more than 10 years before diagnosis were disturbance of color vision, constipation, anxiety, and symptoms of possible RBD, followed by other signs of autonomic dysfunction and reduced sense of smell and shortly before the diagnosis of the PD-slight motor deficits (Table 3, Fig. 1).

**TABLE 3:** Mean duration of prodromal signs in PD patients before the diagnosis of PD

PD-patients	Mean duration of symptoms before the diagnosis of PD in years (SD)
Disturbance of color vision	22.3 (13.6)
Constipation	16.8 (5.7)
Anxiety	13.7 (7.5)
Nightmares	12.6 (7.5)
Involuntarily crying during sleep	12.5 (10)
Involuntarily limbs moving during sleep	11.9 (7.9)
Increased sweating	11.5 (9.6)
Hyposmia/anosmia	11.2 (11.1)
Vivid dreams	11 (6.2)
Moodiness	10.6 (7.7)
Problems with falling asleep	10.4 (8.6)
Depression	10.2 (9.7)
Problems with staying asleep	9.3 (6.6)
Apathy	8.8 (7.1)
Sialorrhoea	8.2 (6.4)
Orthostatic dizziness	8.1 (6.1)
Seborrhoea	5.9 (6.8)
Hypophonia	4.7 (5.2)
Forgetfulness	3.5 (2.9)
Slowing of fine hand movements	3.2 (2.9)
Bradyphrenia	3.1 (2.5)
Problems to recall words	3.1 (2.4)
General bradykinesia	2.9 (1.9)
Dysarthria	2.4 (1.8)
Reduced arm swing	2.2 (2.1)

### Time Course of Prodromal Symptoms in Controls

All signs were listed in the order of their timely occurrence (mean age at onset) from the earliest to the latest (Fig. 1). Controls displayed a similar sequence of symptom manifestation as PD patients but at slightly higher ages, with signs related to RBD and psychiatric complaints occurring earliest, followed by constipation and hyposmia. Similarly, in controls the age of onset for slight motor symptoms was higher than for the PD group.

## Discussion

Our aim was to identify the frequency and timely occurrence of unspecific symptoms supposed to be related to the prodromal phase of PD. In our study population, the mean duration of this phase was 10.6 years. Based on previous neuroimaging and clinical observations, a premotor phase with a probable duration of 10–13 years has been proposed.^8^,^19^

The fact that the investigated prodromal symptoms are more frequently reported in PD patients than in controls underlines the existence of this phase preceding the diagnosis of PD. In our cohort, PD patients reported to have experienced an increasing number of these symptoms with increasing age before the diagnosis of PD was made (Table 2).

The occurrence of these symptoms, which are otherwise unspecific when evaluated singularly, appears to correspond to the neuropathological staging of PD-associated Lewy body (LB) pathology as proposed by Braak et al.^10^ and modified by Przuntek et al.^28^ (Fig. 1).

According to the neuropathological staging of ascending LB pathology, in Braak stage 1 neurodegeneration can be found in the olfactory bulb^29^ and in the lower brainstem comprising among others the dorsal motor nucleus of the vagal nerve and/or the intermediate reticular zone^10^,^28^ as well as in the gastrointestinal tract.^30^,^31^ The expected clinical presentation for this stage would include reduced gastrointestinal motility, i.e., constipation and reduced olfaction, which when regarded as prodromal PD symptoms might occur up to 12 years before the diagnosis of PD. The patients in our cohort reported constipation and hyposmia with a mean of 16.8 and 11.2 years prior to the diagnosis, respectively.

In Braak stage 2, LB pathology and neurodegeneration have been reported to affect the brainstem including noradrenergic and serotonergic nuclei, potentially resulting in mood disorders such as anxiety (up to 20 years before PD diagnosis^17^) or depression (approximately 10 years before motor manifestation^20^) as well as sleep disorders such as RBD, occurring at about 10–12.7 years before the diagnosis of PD in prospective studies.^14^,^32^,^33^ Fitting well with this hypothesis, patients of our cohort reported typical RBD symptoms about 11–12.6 years before the diagnosis of PD as well as symptoms of anxiety (13.7 years) and depression (10.2 years).

With progression to Braak stage 3, neurodegeneration may affect the dopaminergic neurons in the SN, resulting in the presentation of the first, yet unspecific motor symptoms which finally result in the typical clinical presentation allowing PD diagnosis. According to our data, these first minimal motor signs such as slight hypophonia or reduced arm swing may occur about 4.7–2.2 years before the initial diagnosis can be made.

Interestingly, the same chronology of prodromal symptoms was also seen in the controls, however, in a smaller number and at an older age. It may therefore be hypothesized that the occurrence of prodromal symptoms reflects the tenuous border between the normal process of aging and of a beginning neurodegeneration. Recently published neuropathological data suggest that the different stages of LB pathology are passed through by all PD patients but that time of onset and the overall duration of this process may vary by large.^34^ Furthermore, LB pathology corresponding to Braak stages 1 and 2 has been reported in up to 23.7% of clinically healthy individuals,^35^ interestingly similar to our clinical findings also at older ages in relation to PD patients with compatible LB pathology.^36^ Based on these data, it has been speculated that at least some of the clinically healthy subjects with incidental LB might have been in the prodromal phase of PD and could have developed PD at higher age. This assumption can be supported by the relationship of incidental LB disease with RBD,^37^ hyposmia,^38^ and presence of slight extrapyramidal motor symptoms.^39^

Taken together, these findings show that known premotor symptoms occur in a high percentage of PD patients and significantly more frequent than in controls up to 10 to 15 years before motor manifestation. Moreover, our findings suggest that a combination of these symptoms, especially when they occur in a specific chronology may help to identify persons at risk for PD.

As indicated above, some limitations of this study need to be addressed. The main is the retrospective design. For sure, it is difficult for individuals to remember whether they had some specific symptoms and if so for how long they have realized them. Therefore, data cannot be assumed to be entirely exact. Still, the higher prevalence of symptoms prior to the diagnosis PD compared to matched controls is striking as is the timely order of symptom manifestation and its analogy in both groups. However, not all symptoms are perceived immediately at their first occurrence, and problems in assessment occur in both prospectively or retrospectively designed studies. For example, reduced olfaction may develop slowly, and may need a certain amount of impairment before being realized by the patient, whereas RBD could be noticed far earlier leading to a misinterpretation of timely succession, which may also be reflected by our data. It is also important to take into account, that the retrospective recall of the time of onset of specific symptoms after many years can only be an estimation. Therefore, some variation of the time of onset derived from the retrospective recall, and the actual occurrence of sings of neurodegeneration must be considered.

In PD patients a time span of symptoms occurrence to time of diagnosis was used, in controls a span of occurrence to interview. This difference in calculation endpoints needs to be accepted, as there is no time of diagnosis in controls.

Another limitation is that the interview was performed by telephone, without visual contact to the interviewed persons. Hence, a lack of concentration might not have been recognized by the investigator, which may have lead to some bias to data acquisition. On the other hand, all participants were invited beforehand in written form therefore had enough time to prepare for the interview situation in their familiar surroundings. Moreover, the interview situation was exactly the same for patients and controls—therefore, possible shortcomings of the interview technique apply to both groups investigated. However, the lack of validation of the questionnaire needs to be considered, as this assessment tool had been designed for this study. However, it was not meant to be a questionnaire, therefore sensitivity and specificity for the included questions has not been evaluated. The main purpose of this worksheet was to establish comparable interview situations for all participants for acquisition of patients' history information. To our knowledge, to date, no validated questionnaire is available for assessment of putative prodromal PD symptoms in patients and controls. We hope, that the results of this study may help in the definition of potentially valuable items to be included in such a questionnaire.

Further bias to the data may arise from the selection of interview questions, since for example not all potential prodromal signs such as pain or erectile or urinary dysfunction have been included.

Increasing effort is being put in neuroprotective therapies. Therefore, additional diagnostic instruments to facilitate a very early diagnosis of PD are of great importance. A summary of the most probable risk factors reviewed in one questionnaire might be such a tool, which could be easily applicable in patients at first contact in the clinical setting as well as in prospective investigations.
